# *Ginkgo biloba’*s footprint of dynamic Pleistocene history dates back only 390,000 years ago

**DOI:** 10.1186/s12864-018-4673-2

**Published:** 2018-04-27

**Authors:** Nora Hohmann, Eva M. Wolf, Philippe Rigault, Wenbin Zhou, Markus Kiefer, Yunpeng Zhao, Cheng-Xin Fu, Marcus A. Koch

**Affiliations:** 1 Center for Organismal Studies (COS) Heidelberg/Botanic Garden and Herbarium Heidelberg (HEID), University of Heidelberg, Im Neuenheimer Feld 345, D-69120 Heidelberg, Germany; 2 Present address: Department of Environmental Sciences, Botany, University of Basel, Schönbeinstrasse 6, CH-4056 Basel, Switzerland; 3 The Key Laboratory of Conservation Biology for Endangered Wildlife of the Ministry of Education, College of Life Sciences, Zhejiang University, Hangzhou, 310058 China; 4 GYDLE Inc., 1135 Grande Allée Ouest, Suite 220, QC, Québec G1S 1E7 Canada

**Keywords:** Evolutionary history, Phylogenomics, *Ginkgo biloba*, Pleistocene

## Abstract

**Background:**

At the end of the Pliocene and the beginning of Pleistocene glaciation and deglaciation cycles *Ginkgo biloba* went extinct all over the world, and only few populations remained in China in relict areas serving as sanctuary for Tertiary relict trees. Yet the status of these regions as refuge areas with naturally existing populations has been proven not earlier than one decade ago. Herein we elaborated the hypothesis that during the Pleistocene cooling periods *G. biloba* expanded its distribution range in China repeatedly. Whole plastid genomes were sequenced, assembled and annotated, and sequence data was analyzed in a phylogenetic framework of the entire gymnosperms to establish a robust spatio-temporal framework for gymnosperms and in particular for *G. biloba* Pleistocene evolutionary history.

**Results:**

Using a phylogenetic approach, we identified that Ginkgoatae stem group age is about 325 million years, whereas crown group radiation of extant *Ginkgo* started not earlier than 390,000 years ago. During repeated warming phases, *Gingko* populations were separated and isolated by contraction of distribution range and retreated into mountainous regions serving as refuge for warm-temperate deciduous forests. Diversification and phylogenetic splits correlate with the onset of cooling phases when *Ginkgo* expanded its distribution range and gene pools merged.

**Conclusions:**

Analysis of whole plastid genome sequence data representing the entire spatio-temporal genetic variation of wild extant *Ginkgo* populations revealed the deepest temporal footprint dating back to approximately 390,000 years ago. Present-day directional West-East admixture of genetic diversity is shown to be the result of pronounced effects of the last cooling period. Our evolutionary framework will serve as a conceptual roadmap for forthcoming genomic sequence data, which can then provide deep insights into the demographic history of *Ginkgo*.

**Electronic supplementary material:**

The online version of this article (10.1186/s12864-018-4673-2) contains supplementary material, which is available to authorized users.

## Background

During the early Permian, approximately 300 million years ago (mya), Ginkgoatae started to evolve into more than 16 different genera. The genus *Ginkgo* first appeared in the middle Jurassic approximately 170 mya [1, 2]. As indicated by fossil evidence, a worldwide northern hemispheric radiation in temperate forests occurred during the Late Mesozoic and early Tertiary period about 65 mya [1]. However, at the end of the Pliocene and the beginning of Pleistocene glaciation and deglaciation cycles *Ginkgo* went extinct all over the world [1], and only few relict population areas remained in China serving as sanctuary for Tertiary relict trees [3].

There is excellent fossil record of Ginkgoatae (see [3, 4] for further references), and therefore it is not surprising that this gymnosperm tree is among the enigmatic “living fossils” fascinating humans for hundreds of years [5]. *Ginkgo* is a long-lived dioecious tree, and the oldest individuals known in China are estimated to be approximately 1000 to 3000 years old [6]. These old *Ginkgo* trees are often close to human settlements and this also is an indication that the tree has always been playing an important role in medicine, food, ornamentation but also in culture and religion (e.g. [7, 8]). Since a first draft genome of *Ginkgo biloba* was published recently [9] more detailed evolutionary and functional studies may be conducted in the near future.

However, some key questions have not been answered yet such as the temporal dynamics of *Ginkgo* evolutionary history setting a baseline for any further future evolutionary analysis. The status of two major geographical regions in China as refuge areas with naturally existing populations has been proven only one decade ago [3], and it is exciting to study now a “living fossil” in its natural environment with other “tertiary relics”, the warm temperate deciduous forests (WTDF). There is also a continuously increasing interest in the evolutionary history of woodland types of East Asia serving as sanctuaries for many relic tree species from various genera such as *Cercidiphyllum*, *Davidia*, *Euptelea*, *Ginkgo*, *Metasequoia*, or *Tetracentron* [10]. The woodlands that are of particular interest and are often considered tertiary relict vegetation and species assemblages, are referred to as (i) warm temperate deciduous (to evergreen) forests, and (ii) subtropical broadleaved evergreen forests. In particular, the evolutionary and Quaternary history of temperate deciduous species in subtropical China remains under debate. While it is accepted that there are several species from deciduous forest that survived in long-term refugial isolation in subtropical China [3, 10–12], it remains controversial whether these relictual populations underwent glacial admixture [13], or whether constituent species populations of these often montane forest habitats remained isolated over (at least) the latest glacial and interglacial periods [14, 15]). Temperate deciduous forests have been shown to possibly have a wider distribution during the last glacial maximum (LGM, 20,000 years ago) compared to the present-day distribution of potential vegetation using modeling approaches, whilst warm-temperate ever-green forests had a more restricted and southern distribution, respectively [14], also indicating (i) long-term refugial isolation of (warm-)temperate evergreen taxa in subtropical China and (ii) ‘cryptic’ glacial survival of (cool-)temperate deciduous forest trees in North China.

In our previous contribution analyzing range dynamics between co-occurring Asian temperate trees from temperate deciduous forests [16], we elaborated on the relevance of three competing hypotheses that may explain the variation in the degree of expansion to former range limits in eastern Asia. The first hypothesis, introduced by Qian & Ricklefs [13], postulates that during glacial periods populations merged and admixed at lower elevations (isolation with admixture). A second and alternative hypothesis argued that these populations remained isolated during glacial as well as interglacial periods (continual isolation) [14]. A third hypothesis is mediating between both [16] and postulates that life history traits can impose restrictions on the range dynamics and population genetic structure of temperate plants (e.g. [17]). Further contentious topics being discussed under these three hypotheses have been summarized by Zhao and colleagues [18]. The various hypotheses were tested for *G. biloba*, and key traits limiting range expansion were hypothesized such as a slow and complex sexual reproductive cycle, large seeds with slowly developing embryos, and largely extinct fruit/seed dispersers. In the same study, the latest split time of *Ginkgo* refuge areas was calculated to approximately 50 thousand years ago and with strong evidence for genetic connectivity prior to the split and asymmetrical gene flow between regions afterwards. However, large 95% HPD (highest posterior density) ranged from 17 to 95 kya, and, therefore, the split could also be placed either in warming periods during the Last Interglacial (LIG, 120-140 kya) or postglacial warming or in the last cooling phase. It appears that west-east directed regional *Ginkgo* populations have not remained genetically isolated during the entire last glaciation [16]. Effective population sizes of the western and eastern refuge areas were small with 416 and 802 individuals, respectively, and may be dated back to the LIG, whereas ancestral effective population size was estimated with 27,225 individuals [16]. Although plastid DNA sequence data based on a limited number of genes indicated deeper divergence patterns than 50 kya [3, 16], no divergence times have been provided so far. Demographic analyses of population genetic structure from nuclear DNA markers (AFLPs; [3, 5] and independently confirmed structure of gene pools using microsatellites [16]) failed to detect deeper spatial demographic patterns.

There are few studies from species from deciduous forests also showing signatures of expansion during cooling phases [10, 19], and taxa from temperate ever-green forests also exhibit patterns of range expansion [20] and show some similarities with *G. biloba* such as a deep east-west differentiation [16]. On the other hand, for few taxa from EBLF a reverse pattern has been reported with shrinkage of distribution during cooling phases such as during the LGM [21, 22] and postglacial range expansion [23].

To further enlighten these processes, we aimed to unravel a high-resolution temporal maternal evolutionary history of *Ginkgo biloba* considering our previous knowledge on distribution, refuge areas and demography. *Ginkgo* is not only a living fossil, but the entire respective lineage of Ginkgoatae has been isolated for more than 300 million years with *G. biloba* being the only remaining living representative [24, 25]. Consequently, phylogenetic and temporal reconstructions within the lineage are challenging because of missing appropriate outgroups.

The main goals of this study were: (1) To reconstruct a reliable phylogenetic tree to define phylogenetic positions among clades found within *G. biloba*. Although there are numerous fossils of *Ginkgo* and its relatives, these fossils are of no use for calibrating a molecular clock, because fossils are missing that can be assigned to the different present-day gene pools and lineages of extant *Ginkgo biloba*. Therefore, we intended (2) to estimate split times for internal nodes among *Ginkgo* genotypes (e.g. crown group age of *G. biloba*) using primary fossil constraints within a gymnosperm-wide phylogenetic analysis. The results were used for secondary calibration of a larger *G. biloba* data set to (3) elaborate on the hypothesis that *G. biloba* expanded repeatedly during cooling periods throughout the late Pleistocene, and that warming periods such as the LIG forced *Ginkgo* into refuge areas. In order to generate sufficient DNA sequence information from coding and non-coding regions we sequenced entire plastid genomes using a genome skimming approach for subsequent assembly and annotation of plastomes.

## Results

### Sequencing and assembly of plastid genomes

With our genome skimming approach we were able to recover almost complete plastid genomes for all 71 samples. Coverage ranged from 9× to 845× with an average of 102×, and was generally higher for those samples with more raw data available (Additional file 1). Likewise, the number of missing base pairs was higher for samples with low coverage; most samples with coverage < 50× had uncovered bases, while few of those with coverage > 50× had missing data. The highest number of uncovered bases was 402, but only four samples had more than 50 bp missing. Overall, 24 genes were affected by missing data and filtering for low coverage, however in most cases only few base pairs were excluded for a single sample. Libraries for two samples (DHAL6 and WCQF5) were prepared using a different kit and sequenced separately, however this did not affect the quality of sequencing.

### Network reconstruction reveals eight genetic clusters among extant *Ginkgo*

**Fig. 1:**
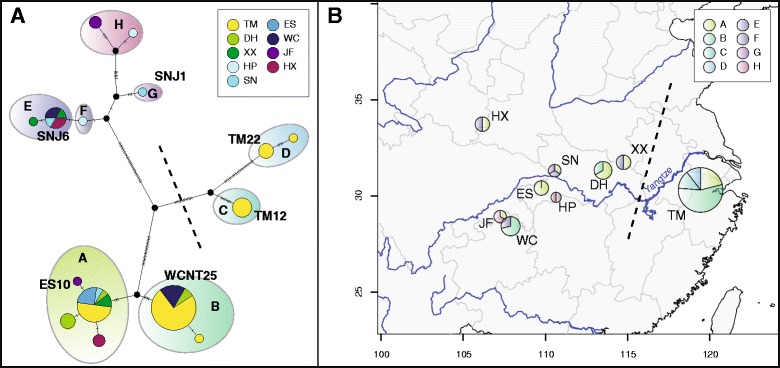
Phylogenetic network reconstruction of 71 *Ginkgo* accessions. **a** TCS network based on complete plastomes. 17 haplotypes were clustered into eight haplotype group (A-H), different colors represent populations of origin. Six sample names denote the samples used in the gymnosperm-wide dataset. Size of haplotype pie charts is proportional to the number of individual sequences assigned to the respective haplotype. **b** Geographic distribution of the eight haplotype groups. Map was created with packages ‘maps’ [62], ‘mapdata’ [63] and ‘mapplots’ [64] in R version 3.2.3 [65] using the ‘worldHires’ map. Detailed geographic coordinates are provided with Additional file 1

The complete plastome alignment of 71 *Ginkgo* accessions comprised 138,921 base pairs (bp) excluding the second copy of the Inverted Repeat (IR) region and indels. A total of 135 polymorphic sites were identified, of which 15 were parsimony uninformative. Consistency index (CI) and retention index (RI) as calculated by PAUP [26] were 95.1% and 99.4%, respectively (CI 93.7% and RI 99.1% calculated without autapomorphies). Network reconstruction revealed 17 individual haplotypes clustered into eight groups (A-H) (Fig. 1a), five of which contained more than one haplotype. Haplotype groups E-H were all closely related with only a few mutational steps separating the respective haplotypes. Haplotype groups A and B were also separated from each other by only a few substitutions, but deep splits were detected between those pairs as well as between groups C and D. It is noteworthy that haplotypes of group C and D were only recovered from the easternmost population in the Tianmu mountains (TM), while haplotypes of group E-H were completely absent in this population (Fig. 1b). These results are in congruence with our earlier studies [3, 16] showing a deep split between Eastern (Tianmu mountains) populations and the populations in Central and Western China (represented by the dashed lines in Fig. 1).

### Gymnosperm-wide phylogenetic reconstruction and divergence time estimation confirmed the cycad-*Ginkgo* sister relationship and revealed Ginkgoatae stem group age of approximately 325 mya

**Fig. 2:**
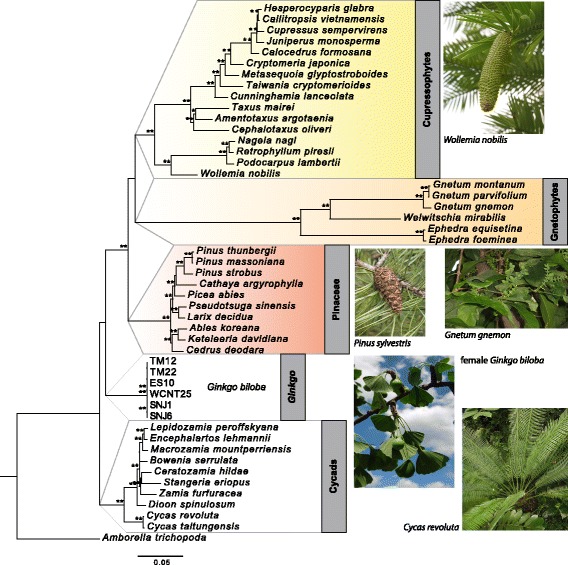
Maximum likelihood phylogenetic reconstruction of *Ginkgo* in a gymnosperm-wide context Bootstrap values from 1000 bootstrap replicates are indicated (** for bootstrap support of 100% and * for support of 95-99%). Bootstrap support was 90% for the split between *Ginkgo* and the group of Pinaceae, Gnetophytes and Cupressophytes. *Amborella trichopoda* (angiosperms) was set as outgroup. Images taken by M.A. Koch

To obtain secondary calibration points and topology information for our 71 *Ginkgo* accessions, we performed phylogenetic reconstruction and divergence time estimation using a subset of our *Ginkgo* samples in a gymnosperm-wide context based on the coding sequence of 78 genes (60,520 bp), including protein coding genes and rRNA genes. Six *Ginkgo* accessions were selected to represent haplotype groups A-E and G. As haplotype groups E-H were closely related, only two representatives were chosen. Over the complete alignment, 17 sites from coding regions were polymorphic within *Ginkgo*, 6 of which were autapomorphies. In total, the gymnosperm-wide alignment comprised 28,905 polymorphic sites, of which 5355 were parsimony uninformative (CI 85.9%, RI 85.1%, excluding autapomorphies 84.7% and 84.0%, respectively). Maximum likelihood (ML) phylogenetic reconstruction using RAxML [27] placed cycads at the base of gymnosperms, and *Ginkgo* as the sister to the clade of (Pinaceae(Gnetophytes,Cupressophytes) (Fig. 2). However, the bootstrap support was low and not significant for the most basal splits between *Ginkgo* and Pinaceae, while it was high for most nodes. The possibility of other topologies can therefore not be excluded.

**Fig. 3:**
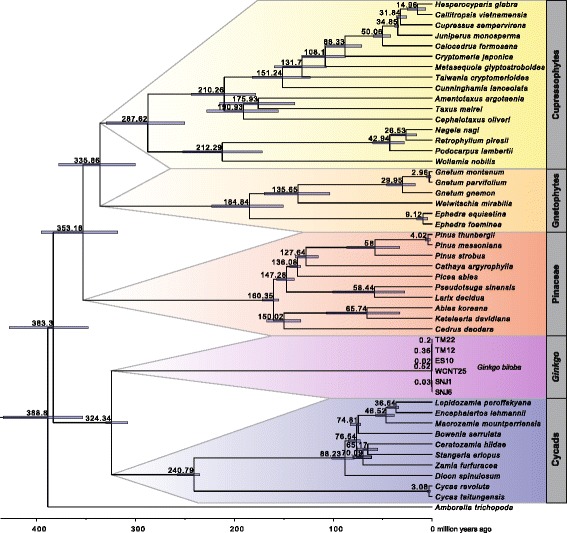
Divergence time estimation of *Ginkgo* in a gymnosperm-wide context. Combined BEAST results from four independent MCMC runs of 5*10^8^ generations each. Calibration was based on 11 fossils (see Additional files 7 and 8). Divergence time estimates are shown with their 95% HPD intervals

The tree topology resulting from BEAST [28] analysis without constraints on tree topology suggested a sister relationship between *Ginkgo* and cycads at the base of the gymnosperms (Fig. 3). This relationship was also supported using the tree topology derived from the RAxML analysis as starting tree for the BEAST analysis. Divergence between *Ginkgo* and cycads was estimated to ~ 325 mya, and the crown age of *Ginkgo* was 0.52 mya using the speciation birth-death model.

**Fig. 4:**
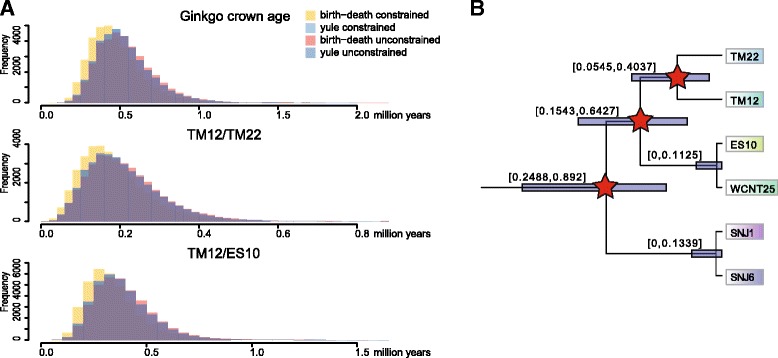
*Ginkgo* secondary calibration extracted from the gymnosperm-wide divergence time estimation. **a** Age distribution from the four different combinations of tree model and topology. *Ginkgo* crown age and the two oldest splits within the species (TM12/TM22 and TM12/ES10) were used for calibration. Displayed are combinations of all four runs for each respective parameter set, excluding burn-in. **b** Magnification of the *Ginkgo* intrageneric nodes from Fig. 3. Upper and lower 95% HPD limits are given. Red stars denote nodes used for secondary calibration in the *Ginkgo* only analysis (Fig. 5)

Constraining the tree topology following the results from ML reconstruction or use of the yule tree model only had a very minor impact on divergence time estimates over all, particularly at younger nodes, and 95% HPD intervals largely overlapped between analyses (Additional file 2). Within the *Ginkgo* clade, the distribution of age estimates overlapped between all four analyses, with the combination of birth-death model and constrained topology being slightly the youngest (Fig. 4a). We used the results from the birth-death unconstrained analysis (Fig. 4a) for secondary calibration at three nodes (Fig. 4b) and selection of the root position for the *Ginkgo*-only analyses. The birth-death model also performed better compared to the yule model in cycads plastome phylogenomics [29].

### *Ginkgo* phylogenetic reconstruction and divergence time estimation revealed successive divergence synchronized with Pleistocene cooling phases

**Fig. 5:**
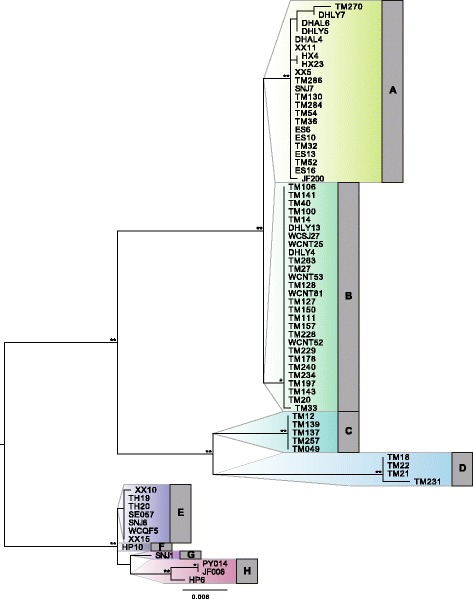
Maximum likelihood phylogenetic reconstruction of 71 *Ginkgo* accessions. Bootstrap values from 1000 bootstrap replicates are indicated (** for bootstrap support of 100% and * for support of 95-99%). The clade containing SNJ1 and SNJ6 (i.e. haplotype groups E-H) was set as outgroup following the analysis in the gymnosperm-wide context

Maximum likelihood tree reconstruction of all 71 *Ginkgo* accessions was consistent with the network analysis and recovered all 17 haplotypes (Fig. 5). Bootstrap support was generally high for both deeper nodes and each of the eight haplotype groups A-H. The position of the root, which was set by selecting the clade comprising haplotype groups E-H as the outgroup following the results from the gymnosperm-wide analysis, was also consistent with midpoint rooting and results from network analysis.

**Fig. 6:**
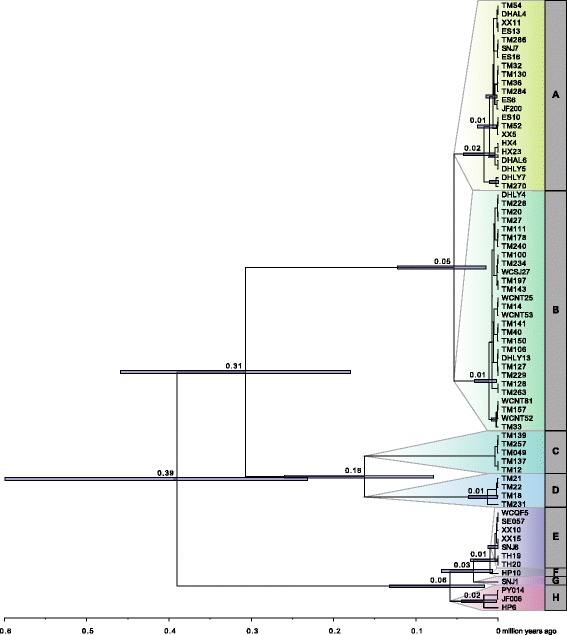
Divergence time estimation of 71 *Ginkgo* accessions. Combined BEAST results from four independent MCMC runs of 1*10^8^ generations each. Calibration was based on secondary calibration derived from the gymnosperm-wide analysis. Divergence time estimates are shown with their 95% HPD intervals for those nodes that represent splits between haplotypes in maximum likelihood reconstruction

Divergence time estimation using the age distributions from the birth-death unconstrained gymnosperm-wide analysis as secondary calibration estimated the crown age of *Ginkgo* to ~ 0.39 mya (95% HPD: 0.22-0.79) (Fig. 6). The subsequent split occurred at 0.31 mya between haplotype groups A-B and C-D. The separation between haplotype groups C and D specific to Tianmu Mountain was much older than that between groups A and B (0.16 vs. 0.05 mya). The initial split within haplotype groups E-H restricted to West China was also dated at 0.06 mya.

**Fig. 7:**
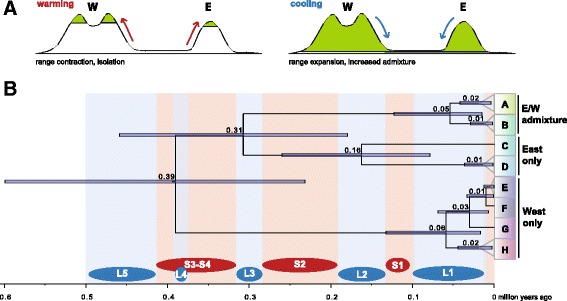
Schematic timeline of *Ginkgo* evolution. **a** Graphical representation of our hypothesis of range contraction and isolation during warming phases and range expansion and admixture during cooling phases. **b** Simplification of divergence time estimations and branching patterns as shown in Fig. 6. Respective warming and cooling phases (S1-S4 and L1-L5 [55]) are indicated

Comparing the occurrence of the various phylogenetic splits with the oscillation of glaciation (L1 to L4) and inter-glaciation/post-glaciation (S1 to S4), we found that all phylogenetic splits were placed into glaciation phases. In other words, any increase in net diversification is linked with cooling phases (Fig. 7).

### Low genetic variation of 35S rDNA loci within and among *Ginkgo* accessions

The alignment of the rDNA sequences showed a very high degree of conservation, as expected. The overall alignment length was 6355 bp including 40 variable sites in total (0.63%) distributed on the different building blocks of the 35S-rDNA locus (Table 1). 36 of these sites are variable within a single individual and represent intra-genomic variation of this high-copy number region.

**Table 1:** Summary statistics of variable sites in the 35S rDNA operon. Intragenomic variables sites include all sites variable within at least one individual, intergenomic variable sites summarize sites with fixed differences (Additional file 3)

	18S rDNA	ITS1	5.8S rDNA	ITS2	25S rDNA	total
Sequence length (bp)	1716	822	163	243	3411	6355
No. intragenomic variable sites	3	6	2	4	21	36
No. intergenomic variable sites		1		2	1	4
Variable ratio (%)	0.175	0.852	1.227	2.47	0.645	0.629

Comparing among accessions there are 34 haplotypes with an overall total nucleotide diversity (π) of 0.00048. Analysis of read data for single individuals revealed that on average intra-genomic nucleotide diversity (π) was 0.00074 with a standard deviation of 0.00024. The alignment with all variable sites is provided with Additional file 3.

## Discussion

### Phylogenetic analysis of gymnosperms and the timeline for *Ginkgo* evolution

Phylogenetic relationships among the five extant seed plant lineages are still not fully resolved and remain uncertain. This includes a hypothesized sister-relationship of cycads to extant angiosperms [30, 31] rather than the more widely accepted monophyly of gymnosperms (e.g. the most recent comprehensive survey: [32]). Within gymnosperms alternative phylogenetic scenarios have been proposed focusing either on the placement of gnetophytes or on the placement of *Ginkgo* compared to remaining gymnosperms. For gnetophyte placement this evolutionary lineage has been considered (i) as the sister to cupressophytes (“gnecup” hypothesis; [25, 33, 34]), (ii) as sister to Pinaceae (“gnepine” hypothesis; [32, 35–37]), or (iii) as the sister to the conifer clade comprising cupressophytes and Pinaceae (“gnetifer” hypothesis, [38]). With respect to the phylogenetic position of *Ginkgo* there is either a basal sister relationship with cycads assumed, setting those two lineages apart from the rest of the gymnosperms (“cycad plus *Ginkgo*” hypothesis; [33, 34, 39, 40]), or a paraphyletic placement among gymnosperms with cycads splitting off first, followed by *Ginkgo* and then followed by the remaining gymnosperms (“*Ginkgo* alone” hypothesis; [24, 25]. From the various phylogenomic analyses it has become evident that *Ginkgo* and cycads are most likely sister lineages and the majority of the most recent studies confirmed this hypothesis. Although our ML analysis was not able to significantly resolve this relationship, BEAST also recognized the cycads-*Ginkgo* sister-relationship when we used a starting tree based on the ML results for the Bayesian (BEAST) analysis. For phylogenetic relationships within gymnosperms “gnecup” and “gnepine” hypotheses are competing with each other, and it seems that the outcome not only varies among studies using the same source of DNA sequence information (e.g. either plastid or nuclear genomes), but also results from the different genomes may remain conflicting. In summary, it seems that plastid data tend to support the “gnecup” hypothesis [25, 33, 34], whereas data from the nuclear genome favor a “gnepine” topology [32, 33]. Our results are also in agreement with these findings. Both ML and BEAST trees based on our plastid genomes favored the “gnecup” hypothesis. The nuclear genome perspective remains unclear. The most recent results from the 1KP project [41] propose the “gnetifer” hypothesis for the nuclear genome perspective, which is in agreement with an earlier transcriptome based analysis focusing on early genome duplications in conifers and other seed plants [42]. However, 1KP data also support the “gnecup” topology for the plastid genome, which is again in agreement with the analysis presented herein. Based on our results we cannot contribute further to these discussions, but having reliably set our results into the framework of most up-to-date findings. This is important for our subsequently performed divergence time estimates and respective careful analyses.

Phylogenetic placement of *Ginkgo* is still under investigation with converging results as discussed above, and similarly the process of the temporal onset of the Ginkgoatae evolutionary lineage is under investigation with converging results. An extensive survey of fossils and justifications for using respective minimum and maximum constraints is provided in [24]. Using minimum/maximum constraints set as 107.7 and 366.8 mya, respectively, they calculated the stem group age of Ginkgoatae with approximately 300 mya. This estimate was confirmed calculating a stem group age of Ginkgoatae with 304.5 mya [43]. For our study, it is important that we were able to confirm consistently this estimate with approximately 324 mya (Fig. 3), and, therefore further divergence time estimates within and among *Ginkgo biloba* clades are feasible. Also tree height (seed plants split/stem group age between present-day angiosperms and gymnosperms) estimated within our study as 388 mya is in the same order of magnitude as demonstrated earlier with 360 mya [24] or 330 mya [43] or the coincidence with the ancestral seed plant Whole Genome Duplication (WGD ξ) 320 mya [44]. A thorough and critical phylogenetic study focusing on divergence time estimates in cycads and testing branching process priors provided crown group estimates for cycad evolution between 274 and 280 mya [29] (240 mya in our study) and a *Ginkgo*-cycad split of 296-323 mya (324 mya in our study).

The crown group age of *Ginkgo* was estimated to 0.39 mya using the total *Ginkgo* SNP dataset (Fig. 6) and demonstrates remarkably that the deepest split among extant *Ginkgo* populations is dating back a few 100 thousands year only and ends abruptly with the third and fourth to last warming (Interglacial) phases (S3-S4, shortly interrupted by a cooling (glaciation) phase L4; Fig. 7; [45]). This indicates that global extinction and decline of *Ginkgo* all across the northern hemisphere (7-10 mya the fossil record disappeared from North America, end of Pliocene of about 2.5 mya from Europe) continued dramatically also in East Asia. Many fossils of *Ginkgo* from the Tertiary and Quaternary are from East Asia [1], however, the youngest *Ginkgo* fossils have been recorded from the late Pliocene and Pleistocene in Japan [1]. In China, there is no record of *Ginkgo* in sediments younger than Eocene. Therefore, it could well be that *Ginkgo* is just an immigrant from Japan to China between 2 and 0.39 mya. Based on limited sampling it seems very likely that old and extant trees in Japan and Korea were introduced from China by humans [3, 5]. Consequently, the evolutionary history of *Ginkgo biloba* before the S3/S4 warming phase remains speculative.

In full agreement with our plastome phylogeny, DNA sequence variation of the entire rDNA locus is extremely low. The highly repetitive 35S ribosomal DNA (rDNA) sequences encoding 18S-5.8S-26S ribosomal RNA very often show high levels of intragenomic uniformity despite frequent occurrence of multiple loci, paralogs and pseudogenes. The homogenization process is known as concerted evolution, but often non-concerted evolution has also been observed frequently associated with hybrid speciation and/or polyploidization [46, 47]. *Ginkgo*’s sister group, the cycads, are characterized by an extraordinary diversity of rDNA repeats [48–50]. This is not only true for the entire cycads, but also for individual cycad species [50]. This feature has been explained by the large genome size (e.g., [51]), dense DNA methylation, frequent methylated cytosine deamination and multiple rDNA loci [50]. Furthermore, long retention times of divergent rDNA paralogs are hypothesized because of the long life-span of several hundred to more than one thousand years. However, *Ginkgo biloba* has a similarly large genome of 11.5 Gbp [9, 52], and it is also a long-lived tree growing to similar ages. Furthermore, whole genome duplications (WGD) have been postulated for both lineages (*Ginkgo* and cycads) recently [9, 53], which can also contribute to rDNA loci duplications. It has been demonstrated that this duplication most likely occurred about 300 mya [53], which correlates well with the stem group age of both lineages. This is, however, in sharp contrast to the finding that rDNA variation in *Ginkgo biloba* described for the entire locus analyzed and spanning 6448 bp was as low as in the annual selfer and angiosperm *Arabidopsis thaliana* [50]. Nucleotide diversity (π) for intragenomic variation within the 18S gene of *Ginkgo biloba* was calculated with less than 0.2% [50]; sequence ID: ERR845259/Illumina HiSeq2000, 170 bp insert size, paired-end sequencing). Our results are fully congruent with this finding, and we did not even observe a higher rate when we compared between individuals considering the entire spatio-temporally structured gene pool. We may explain this at best with the high levels of outbreeding among a dioecious plant and a population structure in habitats largely maintaining genetic connectivity between individuals [16]. Hence, the results from our investigation of the rDNA region are in agreement with the conclusions based on plastid genomes and indicating (a) a very recent evolutionary history, and (b) repeated extensive gene flow and genetic coherence between refuge areas.

### Diversification of extant *Ginkgo* – Modes and tempi

It is obvious that extant *Ginkgo biloba* must also reflect deep signatures of occurrence patterns of Chinese paleo-endemics. In China 20 centers of plant endemism have been characterized [54]. In 35% of these centers *Ginkgo biloba* is found and co-occurs with numerous other paleo-endemics (or even living fossils). Our data indicates that these centers date back several 100,000 years. During the last 400,000 years *Ginkgo biloba* underwent four cooling (“glaciation”) cycles, herein named L4 to L1 [55]. For any of these cooling phases we observed respective phylogenetic splits. In contrast, no splits as indication for diversification were found during any of the warming phases S4 to S1 (Fig. 7). We interpret this pattern as a result of range expansion and increased population size during cooling phases. This increased the likelihood for new haplotype lineages to evolve and persist in a larger gene pool. With the onset of warming phases, the distribution range of *Ginkgo biloba* shrank, genetic drift increased and plastid haplotypes kept separated in refugia while small effective population sizes reduced the chance for new haplotypes to persist.

The temporally deep west–east dichotomous regional genetic structure that we observe in *Ginkgo* is apparent in numerous other temperate woody and herbaceous woodland plants from eastern Asia (*Taxus wallichiana*, *Quercus variabilis*, *Kalopanax septemlobus*, *Schima superba*, *Bubleurum longiradiatum* [12, 18, 19, 56]). Among all of these taxa, the ages of the divisions vary substantially, from 1.45 mya and 1.14 mya in *Q. variabilis* and *B. longiradiatum*, respectively, to 0.28–0.74 mya and 0.61 mya in *K. septemlobus* and *S. superba*, respectively. These examples show that the same large-scale biogeographical pattern evolved several times during cooling and warming cycles and it is likely that present-day regional genetic variation integrates footprints of various cycles of past expansion and retraction. Reconstruction of these sequential processes might be further complicated by varying spatial patterns. In case of Eastern Asian forest biota major shifts towards the North and the South occurred during Pleistocene cooling (glaciation)/warming (deglaciation) cycles and may have also largely influenced temperate deciduous forest species. However, in the case of *Ginkgo biloba* this remains open. We hope that our work will stimulate future studies using genome-wide SNP data to elaborate in detail on population demographic history and processes, e.g. by estimating dynamics of effective population sizes along the geological times of the last 400,000 years and correlating fluctuating effective population sizes with alternating cooling and warming phases while testing different coalescent models.

## Conclusion

We shed some light on the early evolutionary history of Ginkgoatae and estimated a stem group age of approximately 325 mya. Analysis of whole plastid genome sequence data representing the entire spatio-temporal genetic variation of wild extant *Ginkgo* populations revealed the deepest temporal footprint of living populations dating back to approximately 390,000 years ago. *Ginkgo biloba* shows significant genetic structure among past refuge areas, and present-day directional West-East admixture of genetic diversity is shown to be the result of pronounced effects of the cooling period during the last glaciation approximately 100 to 20 kya. *Ginkgo* phylogenetic reconstruction and divergence time estimation revealed successive divergence synchronized with the Pleistocene cooling phases, and we hope that our evolutionary framework will serve as a conceptual roadmap for forthcoming genomic experiments providing deeper insights into the demographic history of contemporary *Ginkgo*.

## Methods

### Plant material and sampling strategy

We collected 71 samples of *Ginkgo biloba* from across its native distribution range in China [3, 5]. With no extant close relatives of *Ginkgo* available, we used a dataset of complete chloroplast genomes available from Genbank/ENA, covering all major clades of gymnosperms, to set the evolution of this living fossil into context. *Amborella trichopoda* as a representative of the angiosperms was used as outgroup. In total, we included 43 published chloroplast genomes. For details see Additional files 1 and 4.

### DNA extraction and sequencing

Total genomic DNA was extracted from silica-dried leaf material using the Invisorb Spin Plant Mini Kit (STRATEC Biomedical AG, Birkenfeld, Germany). Initial homogenization of the material was performed with 2.5 mm glass beads in a Precellys® 24 homogenizer (Bertin Technologies, Montigny-le-Brettonneux, France) in two intervals of 15 s at 5000 rpm. Hereafter we followed the extraction steps as given in the manufacturer’s instructions including the optional RNAse digestion step.

DNA samples were checked for sufficient quality using gel electrophoresis and for concentration on a Qubit® 2.0 fluorometer with the Qubit® dsDNA HS Assay Kit (Thermo Fisher Scientific, Waltham, Massachusetts, USA) before libraries were prepared and sequenced at the CellNetworks Deep Sequencing Core Facility (Heidelberg). 100 to 500 ng of starting material were fragmented on a Covaris S2 Instrument. 63 libraries were prepared using the Ovation Ultra Low DR Multiplex kit 1-96 (NuGEN Technologies, Inc., San Carlos, California, U.S.), and two samples with the NEBnext Ultra DNA kit (New England Biolabs, Inc., Ipswich, Massachusetts, U.S.) with insert sizes ranging from 200 to 400 bp. The 63 and two library sets were then sequenced in 100 bp paired-end mode on one lane of an Illumina HiSeq 2000 sequencing system (Illumina, Inc., San Diego, California, U.S.) each. Additionally, we included data of eight samples from Genbank/ENA study PRJNA307658. Raw sequencing reads generated for this study are available at Genbank/ENA under study PRJEB23626.

### Assembly and annotation of plastid genomes

Illumina raw sequencing reads were filtered by trimming adapters and retaining sequence segments of 50 or more consecutive bases with quality scores 20 or higher to produce high-quality (HQ) sequences suitable for mapping and assembly. We assembled the complete plastid genome of each sample by using an initial mapping of HQ sequences to the *Ginkgo biloba* chloroplast sequence AB684440.1 [57], followed by iterative resolution steps combining automated consensus resolution, gap filling, dynamic remapping of all HQ sequences, interactive edition and assembly visualization. Sequence filtering, read mapping, plastome multi-alignments and annotation were performed with NUCLEAR version 3.2.4 (GYDLE Inc., Québec, Canada). Sequence assembly resolution, edition, visualization and polymorphism identification were done with VISION version 2.6.12 (GYDLE Inc., Québec, Canada). The mapping parameters used to recruit sequences into the plastid assembly (first from the common AB684440.1 reference, then from the iteratively-resolved assemblies) were: -l 40 -s 38 -m 1 --min-pct-cov 80. This selected High-scoring Segment Pairs (HSP = gapless local alignment that achieves the requested alignment scores) of 40 bases or more, containing 38 consecutive identities and containing at most 1 mismatch every 40 bases (amounting to 97.5% local similarity) that were combined into alignments (combination of HSPs with possible gaps between them) covering at least 80% of the fragment’s read sequence (on at least one side for paired reads). The resolving phases of the assembly used local realignments with parameters: -l 30 -s 16 -m 3 --min-score-cov 50 (HSP length > = 30, 16 consecutive identities, 90% local similarity, alignment covering 50 or more bases) to capture differences (fixed mutations and indels) within the alignments prior to resolving the assembly sequence. The assembler always keeps track of the mapping score of each read (in particular which mapped reads are perfectly aligned and which are not), therefore regions consistently connected and covered by strongly aligned reads are not subject to mis-assembly influenced by additional divergent reads, such as those representing insertions into the nuclear genome (which in addition to being divergent and unconnected to the main assembly have also much lower coverage).

The finishing step included the determination of exact junction sequences of inverted repeats and the assignment of a consistent starting position for all the plastomes. The average sequence coverage of plastome assemblies was 102× (minimum 9×, maximum 845×), with 58 out of 71 samples covered at 40× or more. Given the extreme similarity of plastome sequences among *Ginkgo* individuals, we performed two additional curation steps to eliminate sequence artifacts that could affect downstream phylogenetic studies. For this, we aligned all complete assemblies together and reviewed all polymorphisms in the context of their supporting aligned reads in the assembly. First, we identified 20 regions, comprising about 490 bases in all samples, where polymorphisms involved long A/T homopolymers with multiple individuals having inconclusive homopolymer length due to Illumina sequencing limitations. We discarded these regions for the network reconstruction of *Ginkgo* samples. Second, among the 34 mutations and indels specific to a single sample, 18 were documented by very low fragment coverage (often a single read) and were rejected due to possible sequencing error. To put the quality of our assemblies in context, the 18 possible artefacts identified in 71 samples can be compared with the sequence AB684440.1, used as our initial reference, which contains 12 indels and 4 single base mutations that are contradicted by all 71 samples, pointing to 16 sequence artefacts in that single sequence. Annotation of CDS, tRNAs and rRNAs in each sample was performed by extracting the nucleotide sequence of these features in the GenBank record of AB684440.1 and then aligning those to each complete plastome. Complete and annotated plastid genome sequences are available at GenBank/ENA under accessions MG922594 to MG922643.

### General analytical strategy

In order to estimate divergence times for our 71 *Ginkgo* samples, we employed a three-step process of analyses. 1) We reconstructed a network from the entire plastome sequence data of all 71 selected accessions spanning the entire distribution range in putative refuge areas and covering all previously defined haplotype groups and respective phylogenetic splits. 2) Based on the network, we selected a subset of accessions to be included in a gymnosperm-wide analysis using *Amborella* (angiosperms) as the outgroup. This analysis provided us with relationships within *Ginkgo* (for subsequent ingroup rooting) and secondary calibration points for *Ginkgo biloba*, because there is no fossil data available for calibrating the entire Ginkgoatae lineage. The deep divergence in this analysis spanning several hundred million years required us to focus on coding regions only. 3) Based on the results of (2), we expanded the analysis of (1) to phylogenetic reconstructions and BEAST divergence time estimation using the entire *Ginkgo* plastome dataset (with many more synapomorphic mutations) and including respective topological constraints and secondary divergence time calibration points. Among the various steps we cross-validated results comparing tree topologies and divergence times within our study using different models and among the various published studies.

### Step 1: Alignment and network reconstruction of *Ginkgo* samples

All 71 *Ginkgo* plastid genomes were aligned with MAFFT v7.017 [58] as implemented in Geneious v7.1.7 (Biomatters Ltd., Auckland, New Zealand), using the FFT-NS-ix1000 algorithm (200 PAM/k = 2 scoring matrix, gap open penalty = 1.53, offset value = 0.123). Subsequently, the alignment was partitioned into alignment blocks of single exons, introns and intergenic regions. Regions of low alignment quality were excluded using Gblocks v0.91b [59] using the following settings: no gaps were allowed, with minimum block length set to 1 bp. The second copy of the inverted repeat was excluded from further analyses, resulting in a total of 138,921 bp of sequence data. To account for rate heterogeneity among genes, the dataset was partitioned into subsets of genes evolving under the same evolutionary model and with similar substitution rates in PartitionFinder version 2.1.1 [60]. Bayesian information criterion (BIC) was used for model selection, and branch lengths between partitions were allowed to be unlinked. We tested only for partitioning by gene, and only for models implemented in BEAST. We found that our data was best run as a single partition with the GTR + Γ + I model of evolution. A TCS network [61] was reconstructed using PopART (http://popart.otago.ac.nz). A haplotype map was drawn with packages ‘maps’ [62], ‘mapdata’ [63] and ‘mapplots’ [64] in R version 3.2.3 [65] using the ‘worldHires’ map. Consistency index and retention index were calculated in PAUP* version 4.0b10 [26]. The complete alignment and PartitionFinder file as well as results are provided in Additional file 5.

### Step 2: Alignment, phylogenetic reconstruction and divergence time estimations of the gymnosperm-wide dataset

To estimate *Ginkgo* crown age, we selected samples from six different haplotypes (indicated in Fig. 1) and combined them with gymnosperm plastid genome data. Because of high divergence between the included species only coding sequences were used for phylogenetic reconstruction and divergence time estimation of gymnosperms, and subsequently aligned in MAFFT with the same settings as above. Introns were excluded, as were start and stop codons in protein coding genes because of their tendency for higher levels of homoplasy. The final alignment blocks were realigned using MAFFT and manually inspected before they were subjected to an automated alignment quality control using Gblocks with the following settings: minimum number of sequences for conserved and flanking positions was set to 30 (60%), allowed gap number was set to half, and minimum block length was set to 2. Genes were excluded if they were not annotated in a majority of samples (e.g. *rps*16, *psa*M, *ycf*12). The final dataset contained the coding regions of 78 genes (listed in Additional file 5), including four rRNAs, with a total alignment length of 60,520 bp. PartitionFinder was run to identify alignment blocks evolving under the same model and with a similar substitution rate. This resulted in five partitions, each of them evolving under the GTR + Γ + I model. Alignment as well as PartitionFinder file and results are provided in Additional file 6. RAxML version 8.2.3 [27] was used to reconstruct a maximum likelihood (ML) phylogenetic tree. A rapid bootstrap analysis and subsequent ML search was conducted with 1000 bootstrap replicates. The partitioned dataset with five partitions as detected in PartitionFinder was used as input, with GTR + Γ + I as nucleotide substitution model, and *Amborella* set as outgroup. The resulting phylogenetic reconstruction was displayed using FigTree version 1.4.1 [28].

BEAST version 1.7.5 [28] was used to estimate divergence times based on the five data partitions described above. As suggested by PartitionFinder, GTR + Γ + I was used as substitution model for all partitions with four gamma categories. The uncorrelated lognormal relaxed clock was used. We used the RAxML generated ML tree as starting tree after converting branch lengths to obtain a chronogram in R with package ape version 4.1 [66]. For fossil calibration we followed three recent studies on divergence times and divergence time estimation in land plants [43], in gymnosperms [25] and cycads in particular [29]. Fossil ages and their placement for calibration are listed in Additional file 7 and displayed in Additional file 8. To constrain the root height of the tree we used secondary calibration, as no fossil was available for this node. By using a lognormal distribution with offset 249 mya, log(mean) 4.4 and log(stdev) 0.14 we matched the mean and 95% HPD confidence intervals for the split of angiosperms and gymnosperms as estimated in [43]. We used uniform prior distributions for all fossil calibration points with maximum age 330 mya corresponding to the mean of the root height. Significant difference in estimated divergence times for cycads were reported when using different tree models [29], therefore we ran BEAST with both the yule [67] and the speciation: birth-death process [68] model. Additionally, since BEAST resulted in a slightly different tree topology compared to the RAxML results, namely having Cycads and G*inkgo* as sister to a clade of Pinaceae+(Gnetophytes+Cupressophytes) instead of *Ginkgo* being the sole sister group to this clade, we ran BEAST using two different tree topologies. Monophyly constraints were applied either only to all gymnosperms to set *Amborella* as outgroup (unconstrained datasets) or additionally to (Cupressaceae+Gnetales+Pinaceae+*Ginkgo*) to replicate the tree topology from RAxML with cycads as sister to all other gymnosperms. Altogether we analyzed 16 BEAST runs, as both the unconstrained and constrained dataset were run with both tree models four times each. MCMC chain length was 500 million generations, sampling parameters every 50,000 states, thus resulting in 2 billion generations per parameter set. Convergence of the chains as well as ESS values were evaluated in Tracer version 1.6.0 [28]. Output was combined in LogCombiner version 1.7.5 [28], discarding the first 10% of every run as burn in, and subsequently annotated in TreeAnnotator version 1.7.5 [28] using median heights on a maximum clade credibility tree. FigTree was used to visualize the tree and 95% HPD confidence intervals of node ages.

### Phylogenetic reconstruction and divergence time estimations within *Ginkgo*

A maximum likelihood phylogenetic tree of all 71 *Ginkgo* samples was reconstructed with RAxML. Following the results from the gymnosperm-wide BEAST analysis and network reconstruction, the clade containing samples SNJ1 and SNJ6 (haplotypes E-H) was set as outgroup. We ran a rapid bootstrap analysis and subsequent ML search with 1000 bootstrap replicates, with substitution model GTR + Γ + I for the complete alignment, and finally displayed the phylogenetic tree using FigTree.

Considering the not significantly different results from different tree models tree topologies of the gymnosperms phylogenetic tree (Fig. 4 and Additional file 2), and also following Condamine et al. [29], we chose the results from the speciation birth-death tree with unconstrained tree topology (i.e. *Ginkgo* as sister to Cycads) for secondary calibration of *Ginkgo*. Three nodes were selected for calibration: The *Ginkgo* crown age and the two oldest splits within the genus (TM12/TM22 and TM12/ES10). To best represent the posterior distributions of estimated ages at these nodes we extracted the age from each sampled tree (excluding burn-in) and fitted lognormal distributions using the package fitdistrplus [69] in R.

Divergence time estimation was conducted in BEAST. Following the results from the gymnosperm wide analysis, all samples from haplotypes E, F, G and H were set as outgroup through monophyly constraints on this clade as well as the clade of haplotypes A, B, C, and D. As suggested by PartitionFinder we set GTR + Γ + I as substitution model, and we used the lognormal relaxed clock as tree model. Lognormal distributions were used for all secondary calibration points, with log(mean) -0.673 and log(stdev) 0.323 for the tree root height, log(mean) -1.644 and log(stdev) 0.458 for split TM12/TM22 and log(mean) -0.992 and log(stdev) 0.348 for split TM12/ES10. Four independent MCMC chains were run for 100 million generations, sampling every 10,000 generations. Convergence of chains and ESS values were checked in Tracer, then the four runs were combined in LogCombiner, discarding the first 10% of each run as burn-in. The median heights were annotated onto the maximum clade credibility tree using TreeAnnotator, and finally this tree was visualized in FigTree.

### Definition of glacial and interglacial periods

In this study we used the global chronostratigraphical correlation table to extract information from Pleistocene cooling and warming phases in China [70]). Here we followed the nomenclature from the Chinese Loess Sequence for the last 500 ky (S0-S5: warming periods; L1-L4: cooling periods). This record is more appropriate to define cooling and warming periods in East Asia/China and thereby characterizing shifts between woodland and steppe vegetation [55].

### Assembly and annotation of the nuclear 35 S rDNA region

59 of the Illumina datasets generated for this study were subjected to de-novo assembly using CLC Genomic Workbench version 6.9 (Qiagen bioinformatics, Qiagen GmbH, Hilden, Germany), with default settings. The resulting contigs were indexed for BLAST using makekblastdb from BLAST 2.2.28+ [71, 72] and searched for similarity to a sequence containing a partial 35S-rDNA-Sequence (partial 18S-rDNA, ITS1, 3.8S-rDNA, ITS2, partial 25S-rDNA, GenBank/ENA accession number EU643829). This resulted in 59 best-matching contiguous sequences containing partial or complete 35S-rDNA sequences, which were reverse-complemented where necessary using revseq in EMBOSS 6.6.0.0 [73]. By sequence similarity and quality checking in CLC Genomic Workbench a high quality complete sequence was chosen (sample TM14) and used as a reference. All samples’ Illumina reads where then mapped to this reference sequence using bwa version 0.7.5a [74]. After quality filtering and deduplication with samtools version 1.6 [75], variants where called using freebayes version 1.1.0-50 [76] and consensus sequences extracted as FASTA with bcftools version 1.6 [75]. The resulting sequences where aligned using Clustal Omega [77], the alignment was checked and trimmed in PhyDE version 0.9971 [78]. Calculations were done using DNASp version 6.10.03 [79], VCFtools version 0.1.15 [80] and Microsoft Excel 2016.

## Additional files

Additional file 1: *Ginkgo* accession table. (XLSX 21 kb)

Additional file 2: Comparison of all BEAST analyses. (PDF 424 kb)

Additional file 3: 35S rDNA alignment as text file. (TXT 535 kb)

Additional file 4: Gymnosperms accession table. (XLSX 11 kb)

Additional file 5: Partition finder file, partitioning scheme and alignment for gymnosperm dataset as text file. (TXT 2902 kb)

Additional file 6: Partition finder file, partitioning scheme and alignment for *Ginkgo* dataset as text file. (TXT 9655 kb)

Additional file 7: Details of fossil calibration. (XLSX 24 kb)

Additional file 8: Placement of fossil calibration on the maximum likelihood tree. (PDF 225 kb)

## Abbreviations

Kya: Thousand years ago
ML: Maximum likelihood
Mya: Million years ago
