# Supplementary material for: Ginkgo biloba’s footprint of dynamic Pleistocene history dates back only 390,000 years ago
Source: BMC Genomics. 2018 Apr 27;19:299. doi: 10.1186/s12864-018-4673-2 (PMC5921299; doi:10.1186/s12864-018-4673-2)
Supplement: Supplementary file 2 — Comparison of all BEAST analyses. (PDF 424 kb) [file 12864_2018_4673_MOESM2_ESM.pdf]

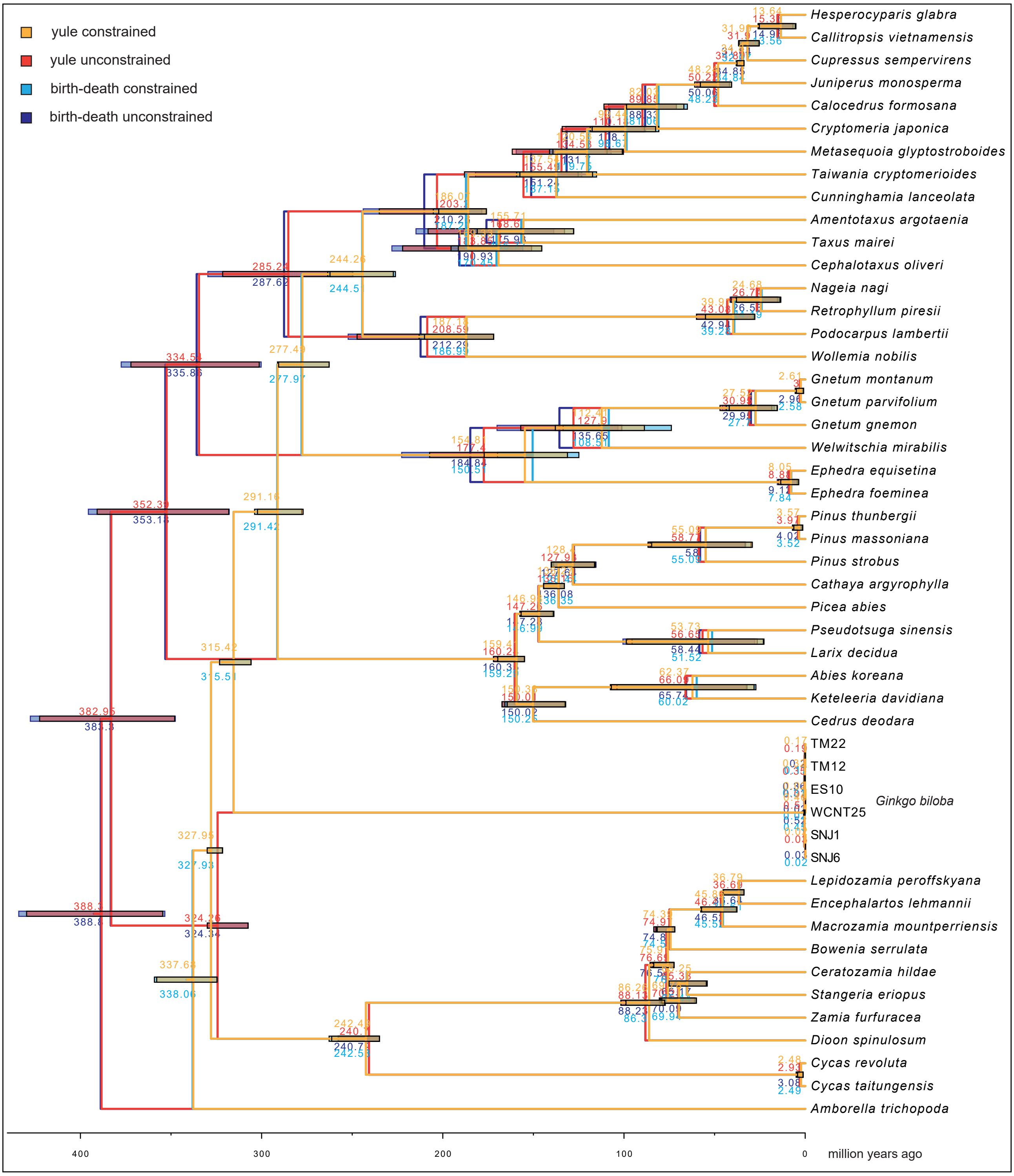

Comparison of four different parameter sets for gymnosperm-wide BEAST analysis. Analyses are based on the same data sets, and differ only in tree model (yule vs. birth-death) and tree topology (no constrainsts vs. constraints to resemble the ML output, i.e. setting Ginkgo as sister to Pinaceae, Gnetophytes and Cupressophytes). Each tree is the result of four independent MCMC runs with $5*10^8$ generations each. 95% HPD intervals are given.
