# Supplementary material for: Ginkgo biloba’s footprint of dynamic Pleistocene history dates back only 390,000 years ago
Source: BMC Genomics. 2018 Apr 27;19:299. doi: 10.1186/s12864-018-4673-2 (PMC5921299; doi:10.1186/s12864-018-4673-2)
Supplement: Supplementary file 8 — Placement of fossil calibration on the maximum likelihood tree. (PDF 225 kb) [file 12864_2018_4673_MOESM8_ESM.pdf]

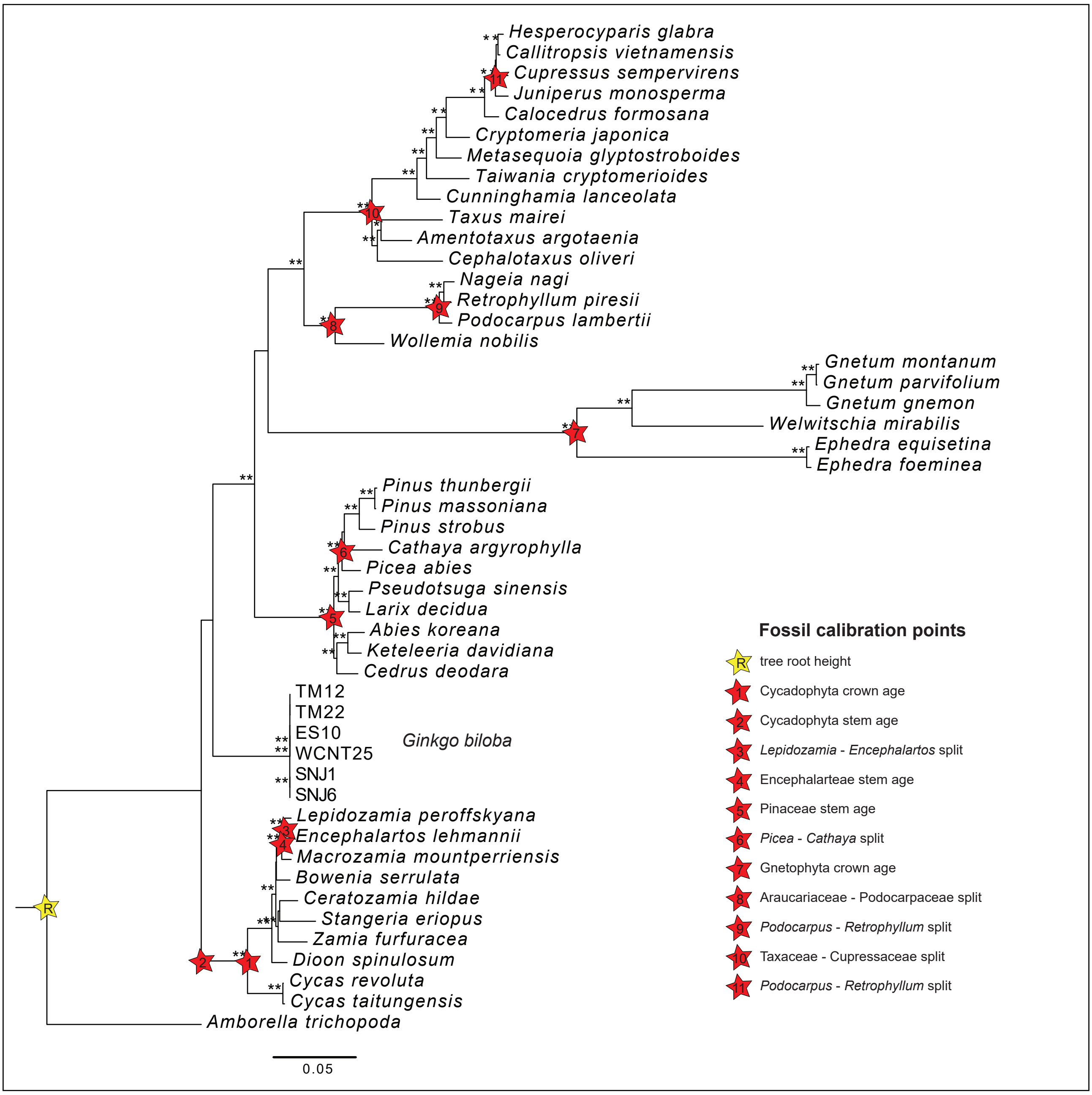

Placement of fossil calibration points for gymnosperm-wide BEAST analysis. Red stars 1-11 represent fossils and their placement following Magallón et al. (2013), Lu et al. (2014) and Condamine et al. (2015), see Supplemental Table 2 for details. Uniform prior distributions were used for all fossil calibration points. Yellow star represents the root node, for which secondary calibration with a lognormal distribution based on Magallón et al. (2013) was used.
